# Molecular Mechanism of Action of Repurposed Drugs and Traditional Chinese Medicine Used for the Treatment of Patients Infected With COVID-19: A Systematic Scoping Review

**DOI:** 10.3389/fphar.2020.585331

**Published:** 2021-02-12

**Authors:** Fui Fui Lem, Fernandes Opook, Dexter Jiunn Herng Lee, Fong Tyng Chee, Fahcina P. Lawson, Su Na Chin

**Affiliations:** ^1^ Clinical Research Centre, Hospital Queen Elizabeth, Ministry of Health Malaysia, Kota Kinabalu, Malaysia; ^2^ Wildlife Health, Genetic and Forensic Laboratory, Kota Kinabalu, Malaysia; ^3^ Biotechnology Research Institute, Universiti Malaysia Sabah, Kota Kinabalu, Malaysia; ^4^ Faculty of Sustainable Agriculture, Universiti Malaysia Sabah, Sandakan, Malaysia; ^5^ School of Medicine, The Johns Hopkins University, Baltimore, MD, United States; ^6^ Faculty of Science and Natural Resources, Universiti Malaysia Sabah, Kota Kinabalu, Malaysia

**Keywords:** COVID-19, alternative medicine, repurposed drugs, SARS-CoV-2, molecular mechanism

## Abstract

**Background:** The emergence of COVID-19 as a pandemic has resulted in the need for urgent development of vaccines and drugs and the conduction of clinical trials to fight the outbreak. Because of the time constraints associated with the development of vaccines and effective drugs, drug repurposing and other alternative treatment methods have been used to treat patients that have been infected by the SARS-CoV-2 virus and have acquired COVID-19.

**Objective:** The objective of this systematic scoping review is to provide an overview of the molecular mechanism of action of repurposed drugs or alternative treatment medicines used to attenuate COVID-19 disease.

**Method:** The research articles or gray literature, including theses, government reports, and official news online, were identified from four databases and one search engine. The full content of a total of 160 articles that fulfilled our inclusion criteria was analyzed and information about six drugs (ritonavir, lopinavir, oseltamivir, remdesivir, favipiravir, and chloroquine) and four Traditional Chinese Medicines (*Shuang Huang Lian Kou Fu Ye*, TCM combination of *Bu Huan Jin Zheng Qi San* and *Da Yuan Yin, Xue Bi Jing Injection*, and *Qing Fei Pai Du Tang*) was extracted.

**Results:** All of the repurposed drugs and complementary medicine that have been used for the treatment of COVID-19 depend on the ability of the drug to inhibit the proliferation of the SARS-CoV-2 virus by binding to enzyme active sites, viral chain termination, or triggering of the molecular pathway, whereas Traditional Chinese Medicine plays a pivotal role in triggering the inflammation pathway, such as the neuraminidase blocker, to fight the SARS-CoV-2 virus.

## Introduction

In December 2019, a novel type of viral pneumonia was discovered in Wuhan, Hubei Province, China. The disease has been officially named “COVID-19 (CoronaVirus Disease 2019)” and the virus has been named SARS-CoV-2 by The International Committee of Taxonomy of Viruses (Gorbalenya et al., 2020). The new coronavirus has rapidly spread among humans all over the world and has led to more than 10 million cases within 6 months. On March 11, 2020, the World Health Organization (WHO) declared COVID-19 a pandemic, which is defined as “worldwide spread of a new disease.” As of July 8, 2020, the number of confirmed cases of COVID-19 globally was 11,669,259, and the total number of deaths has reached 539,906 (4.6%) people (World Health Organization, 2020).

The causation of COVID-19, SARS-CoV-2, is from the family of CoVs, single-stranded RNA viruses that look like a crown under a microscope (coronam is the Latin word for crown) and contain spike glycoproteins on the envelope. It is categorized as a betaCoV, which has an elliptic or round and often pleomorphic form, with a diameter of 60–140 nm. Acute respiratory illness appears to be the most common manifestation of COVID-19 infection. The stages of symptomatic infection range from mild to severe; most infections are not severe (Zhou et al., 2020; Huang et al., 2020; Wang D. et al., 2020):

### Mild Clinical Symptoms

Mild symptoms are present at the time of inoculation and the incubation period, such as malaise, a dry cough, and fever. During this time, the SARS-CoV-2 virus multiplies and primarily establishes residence in the respiratory system. The virus binds to its target through the angiotensin-converting enzyme 2 (ACE2) receptor in human cells (Wan et al., 2020).

### Moderate Clinical Symptoms

Localized inflammation in the lungs and viral multiplication occur in the second stage of the disease. Patients develop a viral pneumonia, with symptoms including fever, cough, and hypoxia. A chest roentgenogram or computerized tomography usually shows bilateral infiltrates or ground glass opacities. Increased lymphopenia and neutrophil-lymphocyte ratio (NLR) are evident in blood tests (Siddiqi and Mehra, 2020).

### Severe Clinical Symptoms

Approximately one out of every six patients transition into the third stage of the illness, which is the most severe and is manifested as an extrapulmonary systemic hyperinflammation syndrome. Systemic inflammation is present during this stage, as well as a decrease in suppressor, helper, and regulatory T cell counts (Qin et al., 2020). Shock, respiratory failure, vasoplegia, and cardiopulmonary collapse are discernible as well as systemic organ involvement and myocarditis.

The latest trend shows that human-to-human spread is the main mode of transmission, which occurs through respiratory droplets resulting from sneezing and coughing. Erosol transmission could also occur in closed areas. Infection might also happen if someone touches a contaminated surface and then touches their own eyes, nose, or mouth (Cascella et al., 2020; van Doremalen et al., 2020). The most frequent source of spread of COVID-19 is people with symptoms; however, the possibility of transmission before symptoms develop, or even from individuals who remain asymptomatic, cannot be excluded. Moreover, the period during which an individual with COVID-19 is infectious is uncertain. The duration of viral shedding is also variable (Cascella et al., 2020; Rothe et al., 2020; Yu et al., 2020). Data and modeling indicate that the use of social distancing is the best way to control this pandemic. Several countries have taken measures such as mobility restrictions, drastic social distancing, school closures, and travel bans, which could significantly disrupt economic and social stability.

At this moment, the therapeutic approaches to handle COVID-19 are only supportive. There is neither a vaccine to prevent infections nor clinically approved antiviral drugs to treat COVID-19. Therefore, the identification of drug treatment options is critical for responding to the pandemic. Clinical trials for vaccines are currently underway in many countries. However, the efficacy of the vaccines, how long immunity will last, or if infection can occur even if a person possesses a high level of antibodies will not be clear for at least 1 year after injection (Callaway, 2020). Furthermore, the safety of the developed vaccines is unknown because laboratory tests are being conducted in parallel with clinical trial phase 1 owing to the emergence of COVID-19 as a pandemic. The unknown efficacy and safety of the vaccines used might cause disease enhancement, by which vaccinated subjects might develop an even more severe form of disease than the subjects that have not been vaccinated, which has been shown in studies of SARS vaccines, in which vaccinated ferrets developed damaging inflammation in their livers after they were infected with the virus (Weingartl et al., 2004).

According to a previous study, disruption of the liver has been reported in patients diagnosed with SARS (Chau et al., 2004) and MERS (Alsaad et al., 2018). This might occur in patients diagnosed with COVID-19 owing to the genome sequence similarity (Zhou et al., 2020) and it has been proven that approximately 2–11% patients had liver comorbidities and 14–53% possess abnormal alanine aminotransferase (ALT) and aspartate aminotransferase (AST) level which occurred more in severe cases of COVID-19 (Zhang et al., 2020). This symptom might be caused by the binding of SARS-CoV-2 to angiotensin-converting enzyme 2 (ACE2) receptor (Wan et al., 2020) to dysregulate liver function and drug hepatotoxicity according to large difference observed from various cohort studies (Zhang et al., 2020). Until now, no drugs have been successfully developed for the control of COVID-19 (Xu et al., 2020); however, numerous efforts are underway worldwide (Lu, 2020).

Drug repurposing has become a model for the rapid development of drugs against infectious diseases, especially in the current global emergency, as it allows the saving of time as well as being a more cost-effective form of drug development. Drug repurposing involves the uncovering of existing therapeutic agents for the treatment of new illnesses or the identification of new therapeutic targets for existing drugs (Pantziarka and André, 2019), including approved, discontinued, shelved, or experimental drugs (Talevi and Bellera, 2020). This strategy could shorten the timeline in drug development because the existing drugs have undergone a scrutinous and extensive process to prove their efficacy and safety to use on humans before marketing surveillance. This strategy has been particularly utilized in oncology and has shown some successes. A notable candidate is aspirin, which is best known for its therapeutic effect in cardiovascular disease but has been shown to have antitumor properties through the suppression of tumors through the inhibition of COX-1 by preventing binding of platelets on tumor cells (Ishida et al., 2016). Prior successes indicate that drug repurposing has a high potential to be the solution for current pandemic while waiting for long-lasting efficacy of vaccination. Therefore, drug repurposing as well as existing alternative medicine could be effective methods for the treatment of patients with COVID-19.

Several previous studies have reviewed drug repurposing for the treatment of COVID-19. However, most reviews have only focused on the mechanism of action of a single drug. In this review, we included 10 medications, comprising six drugs and four complementary medicines. These medications were selected based on their initial successful treatments of COVID-19 patients at the beginning of the COVID-19 outbreak, before it was declared as a pandemic. A few months after the pandemic was declared, the six drugs in this study were officially announced by WHO to be included in a multicountry trial known as SOLIDARITY; the four complementary medicines were included in the national guideline for management of COVID-19 by China, the first country infected by COVID-19. In this comprehensive systematic review of these 10 medications, we discuss the mode of action from a molecular mechanism perspective to attenuate COVID-19 in the human system. By understanding different molecular mechanisms of 10 important drugs instead of a single drug molecular mechanism, researchers could gain a deeper insight of the pivotal genes or mechanisms that should be targeted for future study to ameliorate this pandemic condition, which can also lead to the development of new and effective drugs for the treatment of COVID-19.

## Materials and Methods

A systematic scoping review was conducted from January 1, 2020, until March 18, 2020, by including papers that were published and not published before March 18, 2020, on reported drug repurposing and Traditional Chinese Medicine (TCM) as treatment options for COVID-19 used in patients from different countries to reduce publication bias, increase the comprehensiveness and timeliness of the review, and foster a balanced picture of available evidence (Paez, 2017). The review was performed according to criteria using Preferred Reporting Items for Systematic Reviews (PRISMA) statement (Moher et al., 2009). The list of publications was obtained from the listed databases and search engines: PubMed (https://www.ncbi.nlm.nih.gov/pubmed/), Google Scholar (https://scholar.google.com/), Science Direct (https://www.sciencedirect.com/), and Semantic Scholar (https://www.semanticscholar.org/). Gray literature search such as guidelines, conference papers, theses and dissertations, government reports, research and committee reports, and abstracts were obtained from WHO, Chinese Clinical Trial Registry (http://www.chictr.org.cn/abouten.aspx), U.S National Library of Medicine, China Center for Disease Control and Prevention, China Dissertation Database, China Important Conference Papers Database, and online official news websites. Most of the gray literatures were from China because China is the first country to encounter this disease and provides more data to tackle this disease. Different combinations of the following keywords: NCov-2019, COVID-19, 2019-nCoV, SARS-CoV-2, 2019 novel coronavirus, Wuhan virus, drug repurposing, medication, treatment, traditional Chinese medicine, and alternative and complementary medicine, were used in the literature search through a three-level search strategy based on standardized descriptors defined by the Medical Subject Headings algorithm. A secondary search was based on screening of the reference list of all the relevant studies identified in the direct search. The entire potentially relevant studies were evaluated after title screening to exclude irrelevant information. Only studies that reported the repurposing of drugs and TCMs for COVID-19 and contained information about the structure of the chemical constituents, *in vivo* or *in vitro* studies, case reports, treatment of patients diagnosed with COVID-19, and molecular mechanisms, were extracted and assessed.

## Results

**FIGURE 1:**
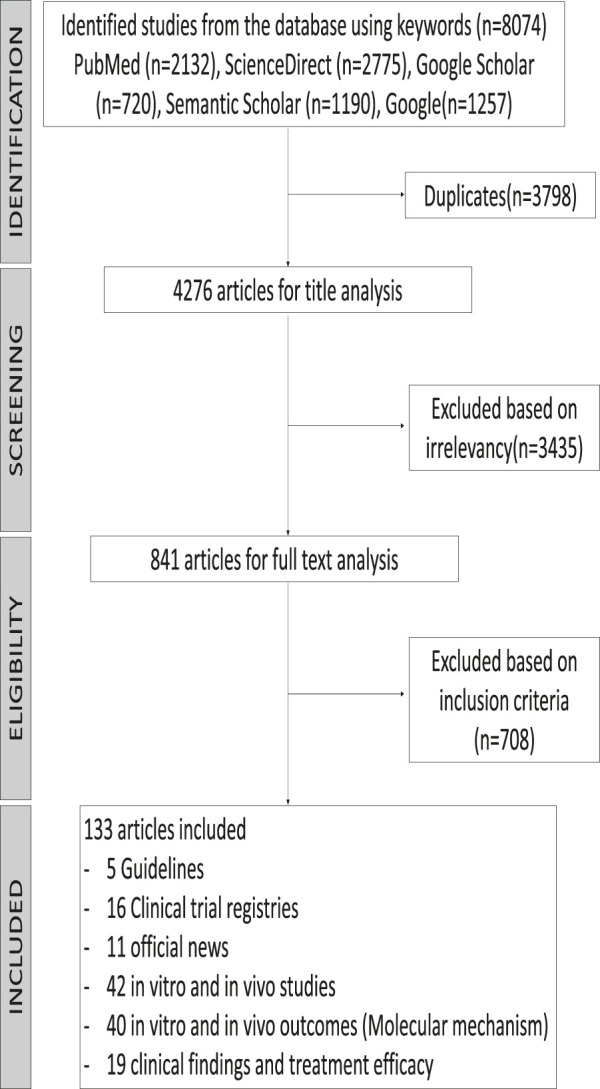
Flow diagram of the systematic search according to the guidelines for Preferred Reporting Items for Systematic Reviews (PRISMA).

The primary search identified 8,074 published and unpublished papers, of which 2,132 were from PubMed, 2,775 from ScienceDirect, 720 from Google Scholar, 1,190 from Semantic Scholar, and 1,257 from Google search engine. A total of 3,798 duplicates were excluded, leaving 4,276 articles for screening by title analysis. 841 eligible published and unpublished papers were identified after excluding 3,435 papers that had a different theme from this systematic scoping review. In conclusion, the content of 841 papers was fully analyzed, of which 681 were excluded according to our exclusion criteria, which was medication that was not administrated as treatment for COVID-19 before March 18, 2020. To establish any differences within a drug class, the drug classes were divided into different subclasses and individual drugs. Repurposed drugs used for treatment of COVID-19 consisted of six drugs (ritonavir, lopinavir, oseltamivir, remdesivir, favipiravir, and chloroquine) and four TCMs (*Shuang Huang Lian Kou Fu Ye*, TCM combination of *Bu Huan Jin Zheng Qi San* and *Da Yuan Yin*, *Xue Bi Jing Injection*, and *Qing Fei Pai Du Tang*). The 133 articles in this review consist of five guidelines, 16 clinical trial registries, 11 from official news, 42 *in vitro* and *in vivo* studies, 40 reports presenting *in vitro* and *in vivo* outcomes, and 19 clinical findings and treatment efficacy. A flowchart of the progressive study selection and numbers at each stage is shown in Figure 1.

## Discussion

**FIGURE 2:**
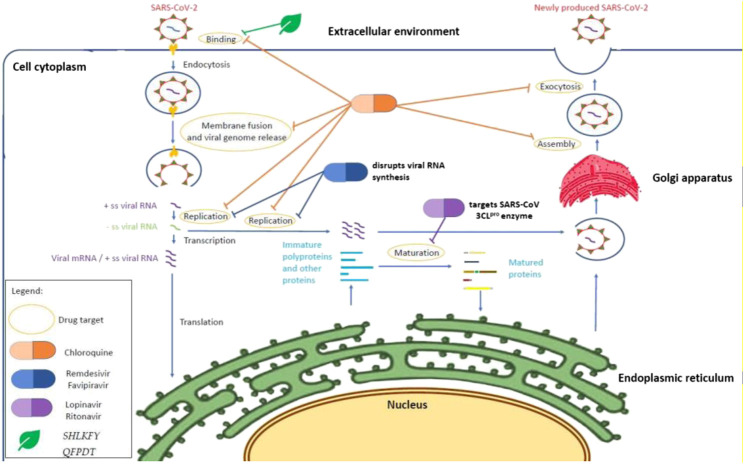
Mechanism of action of repurposing drugs against SARS-CoV-2 within a host cell. Chloroquine inhibits SARS-CoV-2 at the cellular level (Wang M. et al., 2020) when the pH environment is disrupted. HIV protease inhibitors such as lopinavir and ritonavir may demonstrate an antiviral effect through binding to the SARS-CoV 3CL^pro^ enzyme (Nukoolkarn et al., 2008), whereas nucleotides analogues (remdesivir and favipiravir) disrupt the viral RNA synthesis through chain termination (Sangawa et al., 2013; Abdelnabi et al., 2017). On the other hand, *Shuang Huang Lian Kou Fu Ye* and *Qing Fei Pai Du Tang* are suspected to inhibit the binding on angiotensin-converting enzyme (ACE2) owing to the presence of baicalin from *Scutellaria baicalensis* (Yang et al., 2020). The action of oseltamivir and the other two alternative and complementary medicines (Combination of *Bu Huan Jin Zheng Qi San* and *Da Yuan Yin* and *Xue Bi Jing Injection*) remained unknown and are suspected to inhibit the viral neuraminidase according to their previous antiviral effect in influenza virus particles (Mulangu et al., 2019; Harrison, 2020) and altering of the TLR7 signaling pathway (Cheng et al., 2016).

In this systematic scoping review, we provide an overview of previous studies in which existing drugs or Traditional Chinese Medicines (TCMs) have been repurposed for the treatment of COVID-19 and discuss the initial mechanism of action of these drugs from a molecular perspective for the treatment of coronaviruses or other viruses. A summary of the mode of action of all the drugs in this study is shown in Table 1 and Figure 2. WHO reported the launch of a multiarm and multicountry clinical trial of four drugs, remdesivir, lopinavir, and ritonavir (Kaletra), interferon beta, and chloroquine, on March 18, 2020 (Branswell, 2020). Even though our systematic scoping review literature search method covered repurposed drugs used until March 18, 2020, we have covered the majority of repurposed drugs that were used in the SOLIDARITY clinical trial launched by WHO. We did not focus on other drugs that have been recently used for clinical trials because they were not used before the analysis performed for this scoping review. Furthermore, the recent clinical trials have not been multicountry trials; therefore situations such as differences in drug response of the population and adverse drug reactions might exist (Bachtiar and Lee, 2013) and the result cannot represent the efficacy of the drugs for the whole population.

**Table 1:** Treatment of patients during the start of the COVID-19 pandemic.

Type of Drugs	Therapeutic agent	Compounds or components	Mode of action	Reference
Repurposed drugs	Lopinavir Ritonavir	HIV protease inhibitor	Inhibit the SARS-CoV 3CL^pro^ enzyme	Nukoolkarn et al. (2008)
	Oseltamivir	Neuraminidase inhibitor	Inhibits the viral neuraminidase in influenza, however the mechanism in SARS-CoV-2 is still in progress by AbbVie Inc	Harrison, (2020), Mulangu et al. (2019)
	Remdesivir Favipiravir	Nucleotide analogues	Inhibits viral RNA synthesis through chain termination	Sangawa et al. (2013), Abdelnabi et al. (2017)
	Chloroquine	9-Aminoquinoline	Inhibitory effects on SARS-CoV-2 at the cellular level	Wang M. et al. (2020)
Alternative/complementary medicine	Shuang Huang Lian Kou Fu Ye	*Lonicera japonica Thunb.* (chlorogenic acid), *Scutellaria baicalensis Georgi*, (baicalin) and *Forsythia suspensa (Thunb.) Vahl* (forsythoside A)	Inhibit angiotensin-converting enzyme (ACE) by baicalin	Yang et al. (2020)
	Bu Huan Jin Zheng Qi San and Da Yuan Yin	*Atractylodes lancea (Thunb.) DC.*, *Citrus* × *aurantium L.*, *Magnolia officinalis* Rehder and E. H. Wilson, *Agastache rugosa* (Fisch. and C. A. Mey.) *Kuntze*, *Lanxangia tsao-ko* (Crevost and Lemarié) M. F. Newman and Skornick., *Ephedra sinica S.*, *Hansenia weberbaueriana* (Fedde ex H. Wolff) Pimenov and Kljuykov, *Zingiber officinale Roscoe*, and *Areca catechu L.*	Altering TLR7 signaling pathway through regulation of TLR7, MyD88, TNFR6, and IFN-β mRNA in influenza A, yet mechanism of SARS-CoV-2 still needs further investigation	Cheng et al. (2016)
	Xue Bi Jing Injection	*Conioselinum anthriscoides “Chuanxiong*,*” Salvia miltiorrhiza Bunge*, *Paeonia lactiflora Pall*, *Carthamus tinctorius L.*, and *Angelica sinensis (Oliv.) Diels*	Prevent development of a systemic inflammatory response syndrome	Qi et al. (2011)
	Qing Fei Pai Du Tang	*Ephedra sinica S.*, *Glycyrrhiza uralensis Fisch. ex DC.*, *Prunus armeniaca L.*, Gypsum, *Cinnamomum cassia (L.) J. Presl*, *Alisma plantago-aquatica L.*, *Polyporus umbellatus (Pers.) Fries*, *Atractylodes macrocephala Koidz.*, *Poria cocos (Schw.) Wolf*, *Bupleurum chinense DC.*, *Scutellaria baicalensis Georgi*, *Pinellia ternata (Thunb.) Makino*, *Zingiber officinale Roscoe, Aster tataricus L. f.*, *Tussilago farfara L.*, *Iris domestica (L.)* Goldblatt and Mabb., *Asarum sieboldii M.*, *Dioscorea oppositifolia L.*, *Citrus trifoliata L.*, *Citrus* × *aurantium L.*, and *Agastache rugosa* (Fisch. and C. A. Mey.) *Kuntze*.	Reportedly have anti-SARS-CoV activity	Yang et al. (2020), Chen et al. (2017)
			Inhibit angiotensin-converting enzyme (ACE) by baicalin	

### Repurposed Drugs as Drug Candidates in the Solidarity Trial

#### HIV Protease Inhibitors and Anti-Influenza Drugs Used for COVID-19 Treatment

Initially, lopinavir and ritonavir were developed as a standalone antiviral agent for the treatment of HIV infections; however they are combined to obtain a more efficient drug response and sold under the brand name Kaletra (Wishart et al., 2018). Both of the drugs were used for the treatment of HIV-1 and HIV-2 infections through reversible inhibition of the HIV proteases by blocking access to the proteases’ active site, thus preventing the processing of the HIV Gag and Gag-Pol polyproteins (Kempf, 2007). This results in the formation of immature HIV particles that are not infectious. However, the extremely high mutation rate of HIV-1 *in vivo* (Cuevas et al., 2015) has given rise to a strain of HIV-1 that is resistant to ritonavir. The rise of ritonavir-resistant HIV strains has led to the development of more effective drugs for combating HIV infections, one of which is lopinavir. Compared with ritonavir, lopinavir was more effective *in vitro* at a lower amount (17 nM) (Lv et al., 2015); however it undergoes oxidative metabolism by the cytochrome P450 3A4 enzyme in human liver microsomes (van Waterschoot et al., 2010), thus reducing the bioavailability, which is why it was combined with ritonavir for better efficacy.

These drugs were the first repurposed drugs that were used to treat patients diagnosed with COVID-19 (Wipatayotin, 2020). The administration of ritonavir and lopinavir for the treatment of COVID-19 might be because the similarity of the genome sequence to SARS-CoV is approximately 79.6% and it originated from the same genus as SARS-CoV-2, SARS-CoV, and MERS-CoV (*Betacoronavirus*) (Zhou et al., 2020). Therefore, therapies and drugs that have been developed for the treatment of SARS-CoV and MERS-CoV could also be used for the development of COVID-19 drugs, assuming that the mechanism of SARS-CoV-2 is similar to its family members (Yao et al., 2020). Lopinavir/ritonavir have been administered to patients with moderate stage COVID-19 symptoms, which is the second stage of established pulmonary disease and viral multiplication (Siddiqi and Mehra, 2020). Successful treatment of COVID-19 patients with lopinavir and ritonavir has also been reported in India (a 69-year-old male and a 70-year-old female) (Harrison, 2020) and Spain (62-year-old male) (Boyd, 2020).

A molecular dynamics simulation suggested that lopinavir and ritonavir can inhibit the SARS-CoV 3CL^pro^ enzyme by binding to the enzyme’s active site, with neither of them having a higher binding affinity than the other (Nukoolkarn et al., 2008). A recent COVID-19 study revealed that SARS-CoV-2 utilizes the same cell entry method used by SAR-CoV, namely, relying on the ACE2 receptor and priming of the spike protein by TMPRSS2 (Zhang and Yap, 2004). The authors also suggested that antibodies produced against SARS-CoV could potentially be used to combat SARS-CoV-2, albeit at a lower efficiency. Antibodies recovered from recovered COVID-19 patients could be used to combat COVID-19, although only as a preventive measure or in the early stages of the infection (Hoffmann et al., 2020). However, this method was successfully used to treat seriously ill patients in China, with patients showing improvement within 24 h (Bloomberg, 2020).

Even though lopinavir and ritonavir showed their ability to inhibit SAR-CoV during cell entry, a binding analysis showed that half of the lopinavir remained outside of the catalytic site and one of the side benzene side chains of ritonavir might be too long to perfectly fit the substrate binding pocket. This would lead to ritonavir and lopinavir having poor efficacy, and this is reflected in weak *in vitro* activity against SARS-CoV (Zhang and Yap, 2004). In addition, a study conducted on lopinavir’s and ritonavir’s effectiveness against MERS-CoV also revealed that their effectiveness is lower than interferon beta and remdesivir although lopinavir and ritonavir have antiviral activity against MERS-CoV (Sheahan et al., 2020). Another study suggests that both lopinavir and ritonavir have no effect on SARS-CoV replication, with nelfinavir being the only inhibitor that has an effect on SARS-CoV replication (Yamamoto et al., 2004). Despite not manifesting any effect on SARS-CoV replication owing to its low bioavailability, lopinavir possesses antiviral activity against SARS-CoV, with one study suggesting lopinavir has a synergistic effect when used with ribavirin (Chu et al., 2004).

#### Influenza Drug Administered in Combination With an HIV Protease Inhibitor Provides Better Outcome

Oseltamivir is another synthetic prodrug used for the treatment of COVID-19. This drug is initially capable of inhibiting the neuraminidase enzymes on the surface of influenza virus particles. Oseltamivir is administered orally in its prodrug form, oseltamivir phosphate, for the treatment and prophylaxis of influenza A and influenza B infections (Jones et al., 2014). Oseltamivir phosphate is readily absorbed in the gastrointestinal tract, after which it is converted by hepatic esterases into its active form, oseltamivir carboxylase, a competitive inhibitor to neuraminidase found in influenza A and influenza B (Bachtiar and Lee, 2013). It reversibly binds to the active site of the neuraminidase, preventing the neuraminidase from cleaving the sialic acid residues (McNicholl and McNicholl, 2001) found on the surface of the host cell. This prevents the entry of the virus into uninfected cells and the release of newly formed virions from the infected cells and reduces both viral shedding and infectivity of the virus (Bachtiar and Lee, 2013).

On February 18, 2020, a 74-year-old Chinese woman with COVID-19 in Thailand was treated at Rajvitjhi Hospital for COVID-19-related pneumonia with a cocktail of HIV and flu drugs (Yao et al., 2020). The patient was first given ritonavir and lopinavir for 5 days. After failing to show signs of recovery, oseltamivir was administered to relieve the cough and fever symptoms and reduce the severity of these symptoms in the second stage of COVID-19 infection (Siddiqi and Mehra, 2020). This led to a marked improvement in her pneumonia condition in 8–12 h, with the patient testing negative for COVID-19 after 48 h (Mulangu et al., 2019). The drug cocktail was administered for the next 10 days, and subsequent tests for COVID-19 over the next 20 days gave negative results. However, the synergistic effect of the combination of these drugs is unclear because oseltamivir does not inhibit SARS-CoV (Tan et al., 2004) and MERS-CoV like lopinavir and ritonavir (Al-Tawfiq et al., 2014). The repurposing of ritonavir, lopinavir, and oseltamivir for the treatment of COVID-19 is currently being studied by companies such as AbbVie Inc. (Harrison, 2020).

#### Chain Termination of Viral RNA Synthesis by Nucleotide Analogues to Combat COVID-19

**FIGURE 3:**
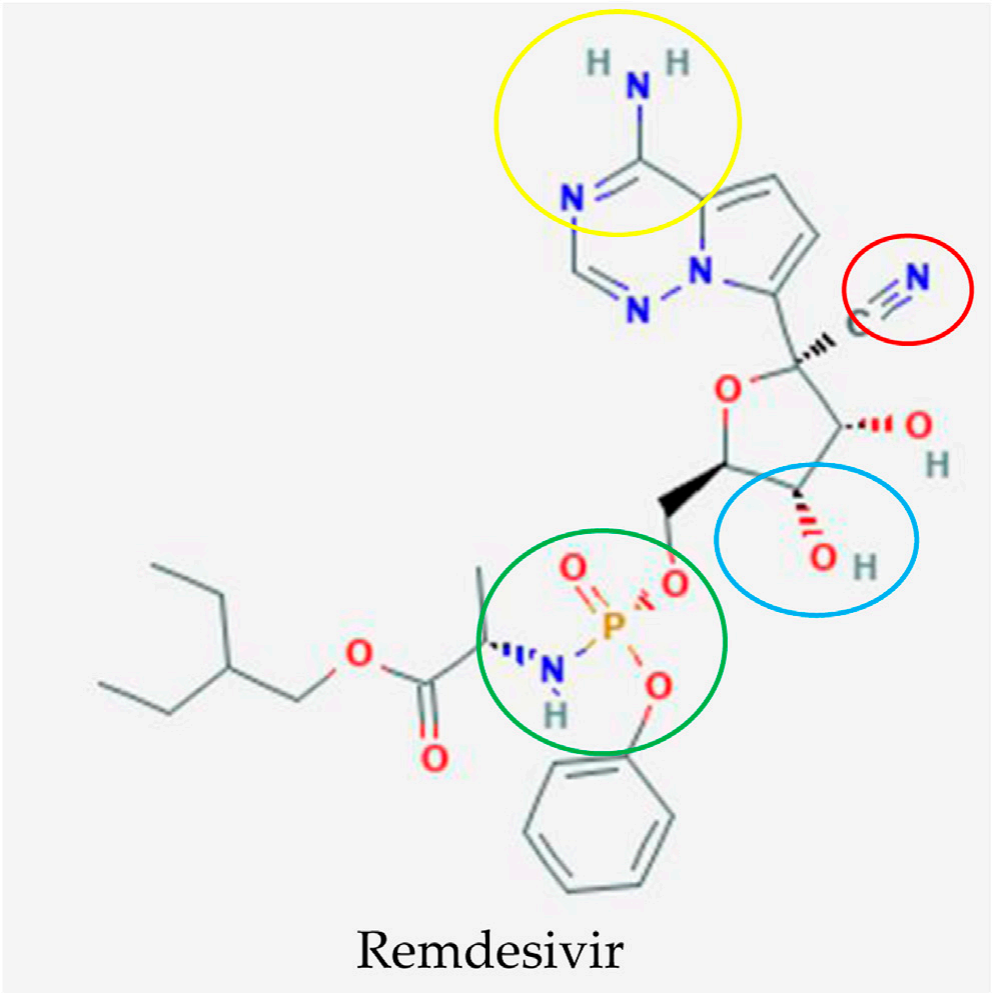
Potential drug candidate to treat COVID-19 manufactured by Gilead Sciences to treat COVID-19, remdesivir (Kim et al., 2019). The addition of 3″ hydroxyl group (blue circle) in remdesivir inhibits the replication of the MERS RNA strand after a few cycles of nucleotide addition, which shields this antiviral drug from removal by coronavirus proofreading enzymes that abolish nucleotide analogs whereas the nitrogens (yellow circle) function as a proton donor and acceptor for a hydrogen bond to a uracil base as an identical binding site of double stranded RNA in adenosine. The phosphate group (green circle) is created as a protecting group or McGuigan ProTide to transport this antiviral compound into cells through phosphorylation, which is identical to a normal nucleotide triphosphate and recognized by polymerases in the cell. The important and unique part for this nucleotide analog is the addition of the 1′ cyano group (red circle) to eliminate the side effect of blockage of the mitochondrial RNA polymerase exhibited in mice.

Remdesivir (RDV; development code GS-5734) is a 1′-cyano-substituted adenosine nucleotide analogue prodrug (Figure 3) that was developed by Gilead Sciences in 2017 as a treatment for Ebola virus infection (Tchesnokov et al., 2019). Several studies have revealed that it has broad-spectrum antiviral activity against RNA viruses such Ebola virus (EBOV), SARS-CoV, MERS-CoV, Marburg, Nipah virus (NiV), respiratory syncytial virus (RSV), and Hendra virus (Dörnemann et al., 2017; Lo et al., 2017; Sheahan et al., 2017).

The antiviral mechanism interferes with the action of viral RNA polymerase, causing delayed chain termination and leading to decreased viral RNA production (Lo et al., 2017; Gordon et al., 2020). Based on an *in vitro* test utilizing primary human lung epithelial cell cultures, remdesivir was potently antiviral against coronaviruses that consisted of Bat-CoVs, zoonotic Bat-CoVs, SARS-CoV, MERS-CoV, and circulating contemporary human-CoVs (Dörnemann et al., 2017; Agostini et al., 2018; Brown et al., 2019). Remdesivir displayed superior antiviral activity *in vivo* in a transgenic mouse with a humanized MERS-CoV receptor (dipeptidyl peptidase 4, hDPP4) and *in vitro* using Calu-3 cells with MERS-nanoluciferase compared with other antiviral drugs such as lopinavir, ritonavir, and interferon beta (Dörnemann et al., 2017). The study stated that both prophylactic and therapeutic remdesivir improved pulmonary function and reduced lung viral loads and severe lung pathology, and an efficacy test of prophylactic and therapeutic remdesivir treatment in a nonhuman primate model of MERS-CoV infection, the rhesus macaque, was performed (de Wit et al., 2020). The induction of clinical disease by MERS-CoV was completely prevented with inhibition of MERS-CoV formation and lung lesion formation after prophylactic treatment initiated 24 h prior to inoculation. The data also strongly suggested a clinical benefit for therapeutic treatment with reduced virus replication and severity of lung lesions.

The improvement of severe lung pathology from *in vitro* and *in vivo* study by Sheahan and colleagues (Yamamoto et al., 2004) might help to improve the illness of patients in severe category of COVID-19, which is manifested as an extrapulmonary systemic hyperinflammation syndrome (Qin et al., 2020), and make remdesivir a drug candidate with a high potential for combating COVID-19. A recent study reported chloroquine and remdesivir effectively inhibit SARS-CoV-2 infection in Vero E6 cells (Wang M. et al., 2020). This drug was even used to treat the first US patient from Washington, who was diagnosed with COVID-19 owing to a pneumonia condition (Holshue et al., 2020). Because the use of remdesivir dramatically improved the patient’s condition, a phase III clinical trial in China, Hong Kong, United States, Singapore, Republic of Korea, and France has been approved to evaluate the efficacy and safety of the drug in patients with COVID-19 (National Institute of Health, 2020). Patients received an initial dose of 200 mg remdesivir followed by a daily dose of 100 mg remdesivir through intravenous infusion in addition to standard of care therapy, whereas patients in the control group received standard of care therapy and the same dose of a remdesivir placebo according to the clinical trial data from U.S National Library of Medicine in 2020.

Favipiravir (FPV; T-705) is another nucleotide analogue developed by Toyama Chemicals for treatment of influenza virus infections (Furuta et al., 2002; Furuta et al., 2013) that has been used to treat COVID-19. It has been demonstrated to have antiviral activity against a wide range of RNA viruses such as norovirus (Rocha-Pereira et al., 2012), Zika virus (Zmurko et al., 2016), foot-and-mouth disease virus (FMDV) (Santos et al., 2006), rabies (Yamada et al., 2016), and Ebola virus (Sissoko et al., 2016; Bixler et al., 2018; Kerber et al., 2019). Similar to remdesivir, favipiravir inhibits viral RNA synthesis through chain termination (Sangawa et al., 2013; Abdelnabi et al., 2017). Favipiravir is metabolized into ribofuranosyl 5′-triphosphate (RTP) and incorporated in the growing RNA strand. The extension of an RNA strand was partially prevented by the incorporation of a single favipiravir-RTP molecule, and the incorporation of two favipiravir-RTP molecules completely blocked further extension (Abdelnabi et al., 2017). This mechanism efficiently inhibits the viral RNA-dependent RNA polymerase function (Smee et al., 2009; Jin et al., 2013; Arias et al., 2014).

To study the effect of favipiravir on COVID-19, favipiravir was approved for the treatment of COVID-19 disease on February 15, 2020. in China. A pilot study of a nonrandomized control trial at The Third People’s Hospital of Shenzhen reported significantly better treatment effects in terms of disease progression and viral clearance compared with lopinavir/ritonavir treatment (Cai et al., 2020). A randomized clinical trial to compare the efficacy and safety of favipiravir and arbidol for the treatment of COVID-19 patients was conducted at three hospitals in China; Zhongnam Hospital of Wuhan University, Leishenshan Hospital, and The Third People’s Hospital of Hubei Province (Chen C. et al., 2020). The trial recruited a total of 240 patients and followed up from Feb 20, 2020, to Mar 12, 2020. Patients in the experimental group received various doses of 1,600–2,400 mg of favipiravir and were compared with patients treated with other multiple antiviral drugs: Kaletra, oseltamivir, and hydroxychloroquine. Favipiravir-treated patients were found to have a higher clinical recovery rate and more effectively reduced incidence of fever and cough (Chen C. et al., 2020) which manifested as mild and moderate symptoms of COVID-19 where during this initial stage, SARS-CoV-2 multiplies and binds to angiotensin-converting enzyme 2 (ACE2) receptor on human cells (Siddiqi and Mehra, 2020; Wan et al., 2020). Other clinical trials of favipiravir monotherapy or combination drug therapy are currently ongoing in China and Thailand to further evaluate the efficacy and safety of favipiravir for the treatment of COVID-19 disease.

#### Chloroquine Antimalarial Drug With a New Effect on COVID-19

**FIGURE 4:**
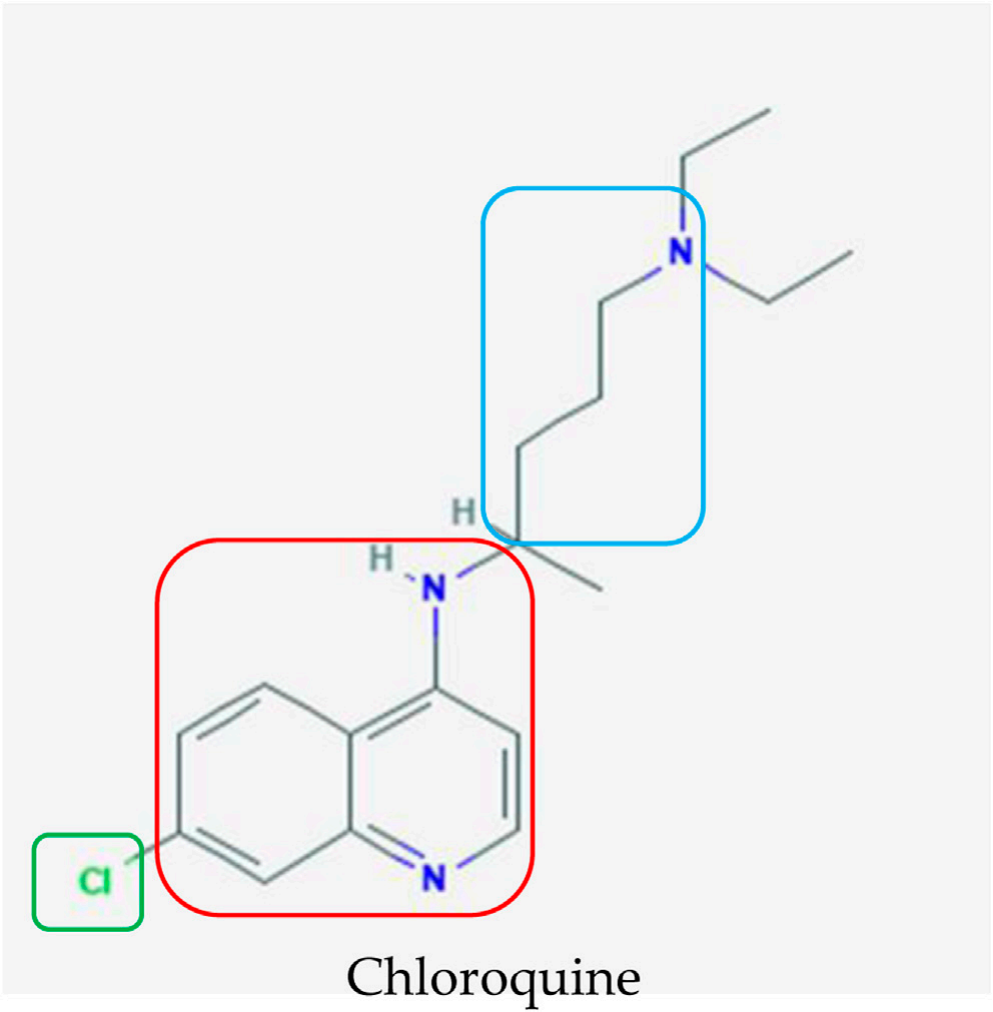
Chemical structure of chloroquine, which was initially used as an antimalarial agent (Kim et al., 2019). The terminal nitrogens and spacer (blue box) were designed to impart parasite resistance whereas the 4-amino quinolone nucleus (red box) and small electron withdrawing group (green box) are pivotal for binding to hematin and halting the formation of hemozoin, respectively, when chloroquine is administered to treat malaria.

In contrast to remdesivir and favipiravir, chloroquine (CQ) is an antimalarial drug considered as one of the drug candidates that exhibit good inhibitory effects on SARS-CoV-2 at the cellular level (Wang D. et al., 2020). Chloroquine is a 9-aminoquinoline that was synthesized in 1934 as an effective substitute for natural quinine used against malaria (Figure 4) (Powell, 1982; Winzeler, 2008; Parhizgar and Tahghighi, 2017). Studies have also reported its versatile antiviral activity against RNA viruses as diverse as the rabies virus, poliovirus, HIV, hepatitis viruses, influenza viruses, and Ebola virus (Kronenberger et al., 1991; Boelaert et al., 2001; Vigerust and McCullers, 2007; Mizui et al., 2010; Dowall et al., 2015; Devaux et al., 2020).

The potential activity of chloroquine against coronaviruses has been demonstrated in different *in vitro* studies. Chloroquine successfully inhibited viral replication of HCoV-229E, SARS-CoV, MERS-CoV, and EBOV in various cell lines (Blau and Holmes, 2001; Savarino et al., 2003; Vincent et al., 2005; Johansen et al., 2013; Madrid et al., 2013; Colson et al., 2020). Conversely, animal studies have revealed mixed results. Treatment with chloroquine showed no significant protection against SARS-CoV and EBOV with reports of high toxicity in mouse and hamster models (Barnard et al., 2006; Falzarano et al., 2015). However, other studies have reported positive results against HCoV-OC43 and EBOV when treated with chloroquine (Keyaerts et al., 2009; Madrid et al., 2013). The contradicting results in animal studies could be owing to the range of doses tested, whereby higher doses could be necessary to produce consistently positive results. However, this could result in a poor outcome owing to an increase in drug-related toxicity. Furthermore, chloroquine may be more effective as a prophylactic treatment owing to its activity during the early stages of a viral cycle, during which it establishes residence in the host through replication of SARS-CoV-2 during the incubation period in patients in the initial stage of the disease, which is a mild condition (Wan et al., 2020).

Recently, Wang M. et al. (2020) reported that the antiviral drugs remdesivir and chloroquine were effective in preventing replication of a clinical isolate of SARS-CoV-2. A clinical trial of over 100 patients also demonstrated that chloroquine phosphate was superior to the control treatment for the inhibition of the exacerbation of pneumonia, promoting a virus negative conversion and shortening the course of the disease, which are symptoms during the severe stage of illness in COVID-19 (Qin et al., 2020). However, the data should be carefully considered before drawing definitive conclusions, because no other results have been published to support this trial. There are a number of clinical trials for the treatment of COVID-19 using CQ registered in the Chinese Clinical Trial Registry (ChiCTR2000029939, ChiCTR2000029935, ChiCTR2000029899, ChiCTR2000029898, ChiCTR2000029868, ChiCTR2000029837, ChiCTR2000029826, ChiCTR2000029803, ChiCTR2000029762, ChiCTR2000029761, ChiCTR2000029760, ChiCTR2000029741, ChiCTR2000029740, ChiCTR2000029609, ChiCTR2000029559, ChiCTR2000029542) (Chinese Clinical Trial Register (ChiCTR), 2020; Kearney, 2020). The requests to conduct these clinical trials have been approved and the findings from chloroquine might explore and investigate the mechanism of action of chloroquine to inhibit SARS-CoV-2.

Even though the trial is still ongoing, there has been a report that long-term usage of chloroquine might contribute to cardiac disorder (Chatre et al., 2018). This drug accumulates in the body and can induce cardiac toxicity if the treatment is longer than 5 years and the cumulative dose is higher than 460 g (Chatre et al., 2018). Despite the fact that the toxicity is rare owing to the variability and nonspecificity, the monitoring of the patients treated with chloroquine to alleviate COVID-19 is essential.

### Alternative and Complementary Medicine Used for the Treatment of COVID-19

#### Traditional Chinese Medicine Can Suppress SARS-CoV-2 *In Vitro*

Complementary medicine has also been used to fight this pandemic disease as an alternative medication. One of the complementary or alternative medicines that have been used is *Shuang Huang Lian Kou Fu Ye*. It was officially used on January 23, 2020, by Beijing Administration of Traditional Chinese Medicine (Morse et al., 2020). It is a Traditional Chinese Medicine (TCM) comprised of three medicinal plants: 375 g of *Lonicera japonica Thunb.,* 375 g of *Scutellaria baicalensis Georgi*, and 750 g of *Forsythia suspensa (Thunb.) Vahl*, according to Pharmacopoeia of People’s Republic of China (Zhang et al., 2013; Xu, 2020)*.*

This complementary medicine was sold out after the announcement made by Wuhan Institute of Virology through articles from Shanghai Institutes for Biological Sciences, CAS, who claimed that this medicine can suppress the SARS-CoV-2 in a cell culture according to a study in collaboration with Shanghai Institute of Materia Medica (Xu, 2020). However, this TCM was not included in the guideline launched by National Health Commission of the People’s Republic of China for prevention of COVID-19 which have been updated to version 7 and known as Guidelines of Diagnosis and Treatment for COVID-19 version 4 report in China for prevention of COVID-19, as reported by China News (Morse et al., 2020; Yang et al., 2020). This is because the findings were limited to an initial laboratory phase and insufficient data were available to confirm that this TCM can suppress SARS-CoV-2. A clinical trial is still essential to verify its efficacy. As a result, Shanghai Public Health Clinical Center and Wuhan Tongji Hospital have initiated a clinical trial on *Shuang Huang Lian Kou Fu Ye* (Yue, 2020). Although there is still insufficient scientific evidence that this TCM can be used to control COVID-19, it is still widely used and the Beijing Administration of Traditional Chinese Medicine claims that it is one of the TCM that can be used to prevent COVID-19. Furthermore, the medicinal plants in this TCM are known for their antiviral function and have been utilized to alleviate influenza and restrain SARS coronavirus. Because both SARS-CoV and SARS-CoV-2 are from the coronaviruses family, the strategies used for the treatment of SARS could be relevant for COVID-19 (Chen Y. et al., 2020).

The antiviral effect is due to the main components from the three plants which are chlorogenic acid, baicalin, and forsythoside A (Shang et al., 2011; Ding et al., 2014; Ding et al., 2017; Zhao et al., 2019). They are found to play a role as neuraminidase blocker (Ding et al., 2017) to inhibit H1N1 and H3N2 from releasing newly formed virus particles from infected cells and by activating the JAK/STAT-1 signaling pathway (Muluye et al., 2014) by inducing IFN-γ production in human CD4^+^ and CD8^+^ T cells and NK cells and by attenuating miR-146a, a pivotal key in the replication of H1N1 and H3N2, by targeting the TNF-receptor-associated factor 6 (TRAF6) (Ding et al., 2014; Li and Wang, 2019; Li et al., 2019; Zhao et al., 2019). These chemical constituents, chlorogenic acid, baicalin, and forsythiaside A, share a common characteristic of being antiviral against influenza; however, their antiviral activity against coronaviruses requires further investigation for further clarification.

### Traditional Chinese Medicine Recommended by China CDC According to Severity of Clinical Symptoms

Based on the Guidelines of Diagnosis and Treatment for COVID-19 Version 5 by the National Health Commission (NHC) of the People’s Republic of China on February 8, 2020, three TCMs have been used depending on the severity of the condition of COVID-19 and symptom differentiation (Yang et al., 2020).

#### Treatment of Mild Clinical Symptoms

For patients that showed mild clinical symptoms such as a dry cough, fatigue, chest tightness, nausea, mild cold fever, or no fever, the TCM consisted of 15 g of *Atractylodes lancea (Thunb.) DC.*, 6 g of *Citrus × aurantium L.*, 10 g of *Magnolia officinalis* Rehder and E. H. Wilson, 9 g of *Agastache rugosa* (Fisch. and C. A. Mey.) *Kuntze*, 6 g of *Lanxangia tsao-ko* (Crevost and Lemarié) M.F.Newman and Skornick., 9 g of *Ephedra sinica S.*, 10 g of *Hansenia weberbaueriana* (Fedde ex H. Wolff) Pimenov and Kljuykov, 9 g of *Zingiber officinale Roscoe*, and 10 g of *Areca catechu L.* (Guo et al., 1989). This decoction is used a combination of the prescribed TCM *Bu Huan Jin Zheng Qi San* and *Da Yuan Yin* adapted from a medical encyclopedia “Gu Jin Yi Tong Da Quan” chapter 76 and “Wen Yi Lun” (Epidemic Diseases) by Wu Youke, respectively, from Dynasty Ming (Wang et al., 2018). Atractylon is a chemical compound present in the chief ingredient, *Atractylodes lancea (Thunb.) DC.*, which attenuated the influenza A virus within 5 days through the TLR7 signaling pathway by upregulating the Toll-like receptor 7 (TLR7), MyD88, tumor necrosis factor receptor-associated factor 6, and IFN-β mRNA expression in the lung tissue of mice infected with influenza A virus (Cheng et al., 2016). The inhibition of coronaviruses by the synergistic effect of this TCM could be similar in action to the inhibition of influenza through triggering of the TLR7 signaling pathway. However, further studies are needed to prove the mechanism of action of this decoction on COVID-19.

#### Treatment of Mild to Severe Clinical Symptoms

Recently, *Qing Fei Pai Du Tang* has been applied for the treatment of patients with any clinical symptoms of COVID-19 ranging from mild to severe cases (incubation period of SARS-CoV-2, viral multiplication, and extrapulmonary systemic hyperinflammation syndrome) (Qin et al., 2020; Siddiqi and Mehra, 2020; Wan et al., 2020) and even used as a preventative medicine for this disease. 1,102 of 1,261 confirmed cases in 10 Chinese provinces were reported to be cured and discharged after the treatment with this TCM (Zhang, 2020). Similarly, the China government has reported that, from 108 patients diagnosed with mild COVID-19 cases, the number of cases that evolved from mild to severe was approximately 10% when given Western medicine alone compared with approximately 4.1% when integrated Chinese and Western medicine treatment was used (Yang et al., 2020). *Qing Fei Pai Du Tang* is comprised of 20 medicinal plants and one mineral: 9 g of *Ephedra sinica S.*, 6 g of *Glycyrrhiza uralensis Fisch. ex DC.*, 9 g of *Prunus armeniaca L.*, 15–30 g of Gypsum, 9 g of *Cinnamomum cassia (L.) J. Presl*, 9 g of *Alisma plantago-aquatica L.*, 9 g of *Polyporus umbellatus* (Pers.) Fries, 9 g of *Atractylodes macrocephala Koidz.*, 15 g of *Poria cocos* (*Schw.*) *Wolf* , 16 g of *Bupleurum chinense DC.*, 6 g of *Scutellaria baicalensis Georgi*, 9 g of *Pinellia ternata (Thunb.) Makino*, 9 g of *Zingiber officinale Roscoe,* 9 g of *Aster tataricus L. f.*, 9 g of *Tussilago farfara L.*, 9 g of *Iris domestica (L.)* Goldblatt and Mabb., 6 g of *Asarum sieboldii M.*, 12 g of *Dioscorea oppositifolia L.*, 6 g of *Citrus trifoliata L.*, 6 g of *Citrus × aurantium L.*, and 9 g of *Agastache rugosa* (Fisch. and C. A. Mey.) *Kuntze*. This TCM is a combination of four combinations of well-known prescribed classic TCMs from Treatise on Cold Damage Diseases, which are *Ma Xing Shi Gan Tang, She Gan Ma Huang tang, Xiao Cai Hu Tang*, and *Wu Ling San* (Zhang et al., 2000; Zhang and Zhang, 2020; Zhang et al., 2020).

*Ma Xing Shi Gan Tang* (MXSGT) is known for antipyretic effects and is commonly used to treat pneumonia, influenza, and other respiratory diseases (Gong, 2018). A systematic review found that the combination of MXSGT with Western medicine significantly increased the effective rate of treatment to treat pneumonia (*p* < 0.00001) (Li et al., 2009) and showed significant improvement (*p* < 0.05) on day 7 of consumption of the decoction besides being effective and safe for the treatment of community-acquired pneumonia (Gong, 2018). This effectiveness is mediated by β2-adrenoceptors on bronchial smooth muscle to inhibit neutrophil from entering the respiratory airway, block acetyl-cholinergic and histaminergic receptor-induced bronchial contraction, and finally reduce neutrophilic inflammation (Kao et al., 2001; Eng et al., 2019). Furthermore, it plays roles in decreasing IL-4, IL-8, and TNF-α, yet increase IFN-γ in a COPD rat model (Zhang et al., 2006). This decoction was also used to regulate the pathogenesis of influenza virus A in infected RAW264.7 cells by the attenuation of LC3, the autophagy marker protein (Li et al., 2019).

A classic decoction used in China and Japan, *She Gan Ma Huang tang* (SGMHT), inhibits mast cells from releasing substances during inflammation, regulates the viscera’s function, and promotes the apoptosis of eosinophils (Zhu, 2014). mRNA expression levels of Th2 cytokines were decreased and associated with Th1 cytokines upregulation and direct attenuation of the pulmonary edema and suppression of the NF-kB pathway through two herbs (*Aster tataricus L. f.* and *Iris domestica (L.)* Goldblatt and Mabb.) from a modified version of SGMHT (Eng et al., 2019). In contrast to the other TCMs mentioned above, the other two TCM formulas (*Xiao Cai Hu Tang* and *Wu Ling San*) function differently and did not exhibit function against acute airway obstruction. *Xiao Cai Hu Tang* (XCHT) is known as a TCM for liver treatment, particularly chronic hepatitis B. This TCM modulates STAT3 expression and indirectly suppresses the hepatitis B virus according to western blot analyses and real time PCR results (Chen et al., 2017). *Wu Ling San* (WLS) has been used to treat impairments of the regulation of body fluid homeostasis in Japan, China, and Korea (Ahn et al., 2012) through affecting the signal transduction pathway such as NF-kB, MAPKs, and HO-1 to demonstrate anti-inflammatory effects like MXSGT to treat pneumonia or respiratory diseases in lipopolysaccharide stimulated macrophages (Oh et al., 2014).

The derived formulation of *Qing Fei Pai Du Tang* from the four combinations of these classic TCM showed its ability to reduce the symptoms of COVID-19 patients by restoring the normal body temperature in 94.6% of 112 patients and stopping coughing in 80.6% of 214 patients (Gu, 2020). As a result, this TCM has been listed as one of the treatment options by the National Health Commission (NHC) of the People’s Republic of China in Guidelines of Diagnosis and Treatment for COVID-19 Version 7 (National Health Commission of the People’s Republic of China, 2020; Yang et al., 2020). The effectiveness of *Qing Fei Pai Du Tang* can reach 97.78% with 1,102 patients being cured from 1,261 patients from 10 districts in China until March 13, 2020 (Yang et al., 2020; Zhang et al., 2020). Until now, none of the cases became severe from mild conditions after the consumption of *Qing Fei Pai Du Tang*. However, any pharmaceutical drugs can induce side effect or cause adverse events and TCM herbs are not an exception (Chow et al., 2019). There is evidence that one of the herbs (*Glycyrrhiza uralensis Fisch.*) used in this concoction possesses hepatoxicity in a clinical trial from an extensive literature review study through standardized causality assessment algorithms, Roussel Uclaf Causality Assessment Method (Chow et al., 2019). As a circumstance, the toxicology of the TCM concoction should be determined to ensure the safety of patients, even though so far there has been no report of side effects caused by the alternative or complementary medicine mentioned in this study.

#### Treatment of Severe Clinical Symptoms

*Xue Bi Jing Injection*, an established TCM in China, has been recommended for severe symptoms of COVID-19 patients (National Health Commission of the People’s Republic of China, 2020; Yang et al., 2020). Unlike other drugs that undergo a conventional clinical trial phase, it proceeded from bedside to bench and finally back to bedside before approval was obtained from China Food and Drug Administration in 2004 (Zhang et al., 2018). *Xue Bi Jing Injection* consists of numerous compounds, including Senkyunolide I, safflor yellow A, paeoniflorin, ferulic acid, galloylpaeoniflorin, anhydrosafflor yellow B, oxypaeoniflorin, caffeic acid, albiflorin, uridine, gallic acid, guanosine, danshensu, protocatechuic aldehyde, and hydroxysafflor yellow A, which are extracted from five medicinal plants, *Conioselinum anthriscoides “Chuanxiong*,” *Salvia miltiorrhiza Bunge, Paeonia lactiflora Pall*, *Carthamus tinctorius L.*, and *Angelica sinensis (Oliv.) Diels* (Gong et al., 2015).

It was initially developed for activating blood circulation to remove blood stasis, cooling the blood, and clearing toxic heat (Zhang et al., 2018). However, this TCM has been used to fight SARS-CoV-2 because of its effectiveness in treating severe pneumonia, the severe stage of COVID-19, by significantly reducing mortality by approximately 15.9% and elevating the improvement of the pneumonia severity index by approximately 60.8% (Zhang et al., 2018). The *Xue Bi Jing Injection* relieved or reduced severe pneumonia by triggering the inflammation pathway through downregulation of TNF-α, IL-6, and IL-8 on the 3rd, 7th, and 14th day after treatment, although it did not significantly influence the release of leptin (Qi et al., 2011). This suggests that an antiendotoxin effect was deployed by halting the release of TNF- α, IL-6, and IL-8, endogenic inflammatory mediators. As a result, blocking the development of a systemic inflammatory response syndrome occurred through the disruption of the inflammation vicious cycle (Qi et al., 2011). This mechanism of action of the *Xue Bi Jing Injection* reduces the severity of pneumonia in COVID-19 patients and lowers the side effects on the organ functions.

These results indicate that TCMs can be used as complementary medicine for the treatment of patients during this pandemic disease; however, the mechanism of action of these TCMs still requires further investigation and validation.

## Conclusion

The treatment of COVID-19 with the six repurposed drugs discussed in this study is dependent on the ability of the drug to inhibit the proliferation by binding to the enzyme active sites, viral chain termination, and triggering of molecular pathways. In contrast to the six drugs, the four TCMs discussed in this review were initially used to treat influenza and SARS by acting as neuraminidase blockers and trigger the inflammation pathway. In this review, we provide a framework for better understanding of the mechanism of action of repurposed drugs and TCMs and their involvement in the molecular pathway for inhibiting viral replication such as SARS or MERS.

Instead of focusing on one drug mechanism of action, we analyzed repurposed drugs that are currently involved in worldwide clinical trials to elucidate their molecular mechanism. The inclusion of gray literature provides more data to better understand the properties and effects of repurposed drugs. However, there are limitations to our findings because the quality of the evidence and risk of bias were not evaluated due to the nature of a scoping review. Furthermore, there is still a lack of concrete evidence for the mechanisms of action of the drugs and their curative effect on COVID-19 because the clinical trial is still ongoing. Further experimental validation is needed to provide more concrete evidence.

Understanding the different molecular mechanisms of these drugs instead of just one drug molecular mechanism can provide insights on the pivotal genes or mechanisms that should be targeted in future studies and lead to the development of effective drugs for the treatment of COVID-19 in the future.

## Data Availability

The data analyzed in this study is subject to the following licenses/restrictions: No restrictions. Requests to access these datasets should be directed to FFL, lemfuifui@moh.gov.my.
